# Spiral Humeral Fracture During Arm Wrestling: A Case Report and Literature Review

**DOI:** 10.7759/cureus.29540

**Published:** 2022-09-24

**Authors:** Muhanand Wael, Mohammad M Mahmoud Jaber, Mazen A Abdullah, Saif Atyani, Abdulmalik Jaber, Nabila H Halta, Mohammad Ghannam

**Affiliations:** 1 Faculty of Medicine, An-Najah National University Hospital, Nablus, PSE; 2 Orthopedic Surgery, An-Najah National University Hospital, Nablus, PSE; 3 Medicine, An-Najah National University Hospital, Nablus, PSE

**Keywords:** physiotherapy, closed fracture reduction, wrestling, humeral fractures, orthopedic

## Abstract

Arm wrestling places an axial pressure load on the humerus with the glenohumeral joint stabilized and the elbow flexed and fixed. This situation can cause humeral shaft fractures. We present a case of humeral shaft fracture in a 22-year-old healthy man following an arm-wrestling challenge. The patient is known to be a bodybuilder and athlete. He presented to our university emergency department with a swollen and tender arm and intact neurovascular structures, reporting that he had recently engaged in arm wrestling with a colleague of similar shape and power. The fracture was treated conservatively based on the patient's informed decision using closed reduction and physiotherapy. He committed to scheduled clinic visits and physical therapy sessions and showed improvement with complete recovery and normal functioning on the thirteenth week.

## Introduction

Arm wrestling is a popular sport where two contestants sit or stand with their hands gripped while trying to pull each other away by positioning a fixed, flexed elbow on a table [1]. Despite arm wrestling's appeal as entertainment and a form of boosting the ego of the winner, the activity is detrimental to participants and should not be considered a professional, competitive sports challenge [2]. Fracture of the humerus following an arm-wrestling match is rare [3]. Arm wrestlers should be aware of the dangers associated with the sport due to the possibility of serious problems. We present a case of humeral shaft fracture in a 22-year-old healthy, athletic man who presented to the emergency department of Al-Najah National University Hospital (Nablus, Palestine), after participating in an arm-wrestling challenge.

## Case presentation

**Figure 1:**
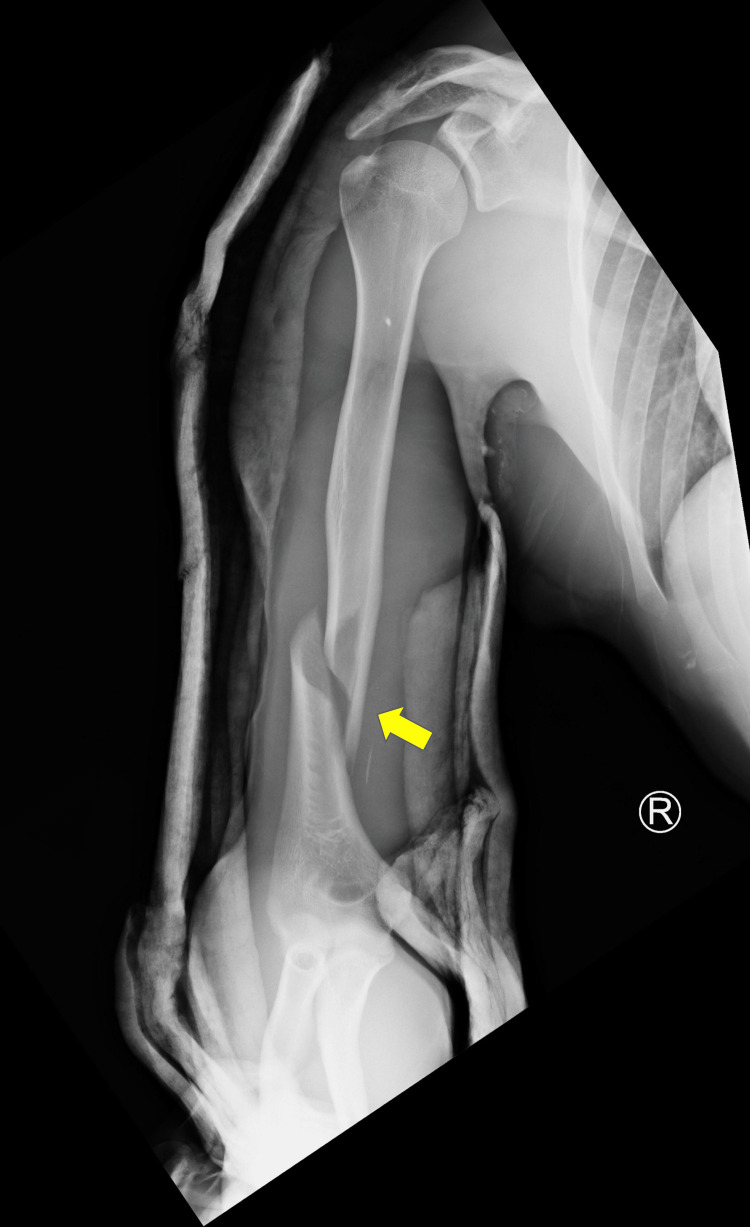
An X-ray on admission showing a distal, one-third spiral humeral fracture (arrow)

A 22-year-old, healthy, athletic, young man presented to our university emergency department with a swollen and painful humeral shaft fracture. The patient is a bodybuilder with good nutritional habits. He reported participating in an arm-wrestling challenge with a colleague of similar shape and power. He reported excruciating pain in his entire arm as it cracked and gave way in the winning phase of the match. We noted a disclosed painful right arm with intact neurovascular structures on examination. An X-ray of the injury revealed a distal, one-third spiral humeral fracture (Figure 1).

Treatment options and consequences in recovery time, quality of life, and all aspects of the management were discussed in detail with the patient. He had immense concerns regarding the operative options and expressed a strong desire for conservative management. We performed a closed reduction in the emergency department and fitted the patient with an immobilizing cast for three weeks. During the third week, the patient was changed to a fiber cast to stabilize the shaft only, allowing free elbow movement.

The patient mentioned stiffness and uncomfortable movements in the elbow that gradually resolved after starting home-based physiotherapy in the fifth week. In the seventh week, the fiber cast was removed. In the eighth week, the patient achieved clinical full recovery and range of motion. The patient was able to return to the gym in the eleventh week.

At his one-year follow-up examination, the patient reported full functional recovery and regular activity with heavy loads during his gym workouts. According to the patient, the event could be largely forgotten, "as if it never happened."

**Figure 2:**
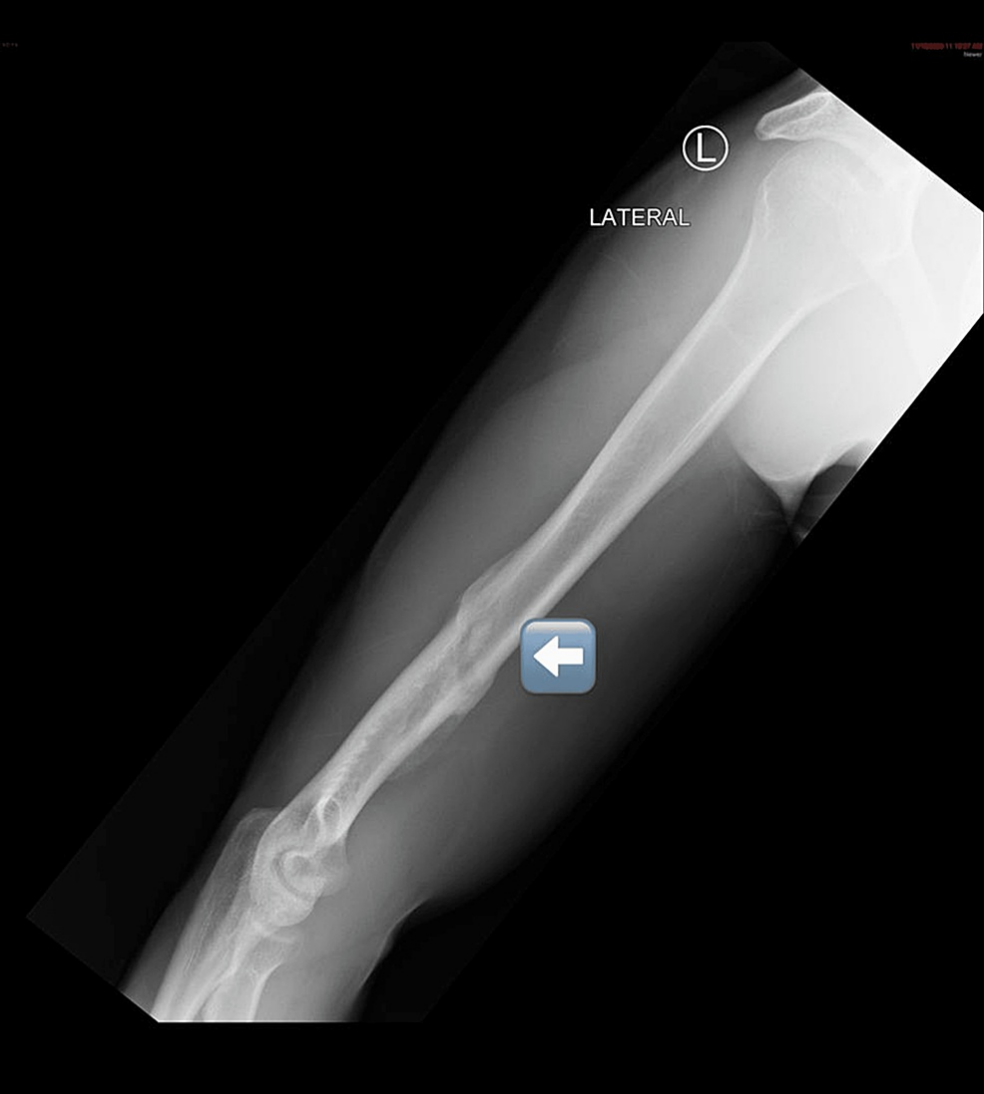
On the tenth-week visit, the X-ray showed well-healed and complete union

## Discussion

Arm wrestling is a risky activity. We conducted a narrative review of the literature concerning arm-wrestling injuries. About 152 cases of arm-wrestling injuries were reported in the literature. The most common injury associated with arm wrestling was spiral fractures of the distal part of the humerus. Torsion and bending loads cause the humerus to fail. In a study, in 23% of the cases, a medial butterfly fragment and radial nerve palsy were both discovered. The skeletally immature arm-wrestler has an increased frequency of medial humeral epicondyle fractures, which differs from the skeletally mature arm-wrestler [4].

The shoulder joint is actively internally rotated against the opponent while the elbow is fixed in flexion, resulting in enormous violent torque forces across the humeral shaft. The bone is more prone to fracture when it is rotated, twisted, and subjected to a lot of axial pressure. The types of fractures that occur at the middle and distal thirds of the humeral shaft are related to the anatomic and materialistic features of the bone [1]. The tension placed on the right and left internally rotate when they change from concentric to oblique, causing immense violent torque pressure to pass over the shaft of the humeral bone. Due to the immediate proximity of the radial nerve to the typical fracture site, 8% to 12% of patients experience radial nerve palsy with humeral shaft fractures [5].

The opponent's strength does not influence the occurrence of humeral shaft fracture [6,7]. Multiple anatomic factors, including bone density, may directly cause or predispose a patient to fractures in arm wrestling. Spiral fractures are potentially the result of bending, axial pressure load compression, and torsional forces applied to the humerus during the match [8]. According to Kruczynski et al., the primary bone stress is due to extreme loads of up to 60 MegaPascals placed 115 mm above the elbow on the posteromedial portion of the humerus [9]. This was determined during simulated arm-wrestling scenarios in a biomechanical evaluation study based on computed tomography (CT) scans of the humerus bone. Due to the strains of the active muscles supporting it, the distal one-third of the humeral shaft was severely loaded during arm wrestling [9]. Pedrazzini et al. reported that the distal part of the humeral shaft has less bone mineral content and a lower ratio from the outer diameter to the inner diameter than the rest of the bone, according to their study using CT scans and bone density testing [10]. Anabolic steroid use causes maladjustment in the bones' cortical thickness and the muscles' power, which may be a causative or predisposing factor for arm-wrestling fractures [1].

Pande et al. reported a case series of six arm-wrestling-related fractures in nonprofessionals and one in a professional. Moreover, the opponents were relatively equal in power, shape, and limb length in each of the fractures [1]. Three patients in the Pande et al. series were treated conservatively, and three were treated with surgical correction. This conflicts with the report from a kinematic and electromyographic study that suggested that the strength of the pectoralis major muscle might offer a participant a winning advantage in the competition [11]. Marko et al. reported a case series in which none of the participants (n=6) experienced neurovascular complications or insults, and similar to Pande et al., half of the patients were treated conservatively and half received surgical correction [12].

Management of humeral fractures may cater to the patient's preferences because most humeral shaft fractures can be treated conservatively using plaster splints, hanging casts, or functional bracing unless operative intervention and fixation are indicated [5]. Conservative treatment is effective, and the primary differences from operative management are the rapid healing and recovery times. However, surgical management would be indicated for neurovascular insult, open fractures, multiple fractures, and pathologic fractures, pending the informed consent of the patient [13]. The most common surgical treatment options are open reduction and internal fixation with plating, minimally invasive plating, and intramedullary nailing or external fixation in severe soft tissue compromise [14,15]. Sarmiento et al. reported a 97% union rate in 922 fracture patients, and 67% were committed to scheduled clinic visits until satisfactory healing was noted [16].

## Conclusions

We described a case of humeral shaft fracture in a 22-year-old, otherwise healthy man following an arm-wrestling challenge. The spiral humeral fracture from arm wrestling does not appear to be related to the participant's position or phase of the match, nor competitor strength and intensity, as in our case. Many factors may cause or predispose a patient's arm to fracture during arm wrestling, which is a risky activity.

## References

[1] Pande K, Nishat N, Afzal S, Ishak L (2021). Humeral shaft fracture sustained during arm wrestling with review of factors contributing to its causation. Malays Orthop J.

[2] Diffrient DS (2019). (Arm) wrestling with masculinity: television, toughness, and the touch of another man’s hand. Men Masc.

[3] Khashaba A (2000). Broken arm wrestler. Br J Sports Med.

[4] Moloney DP, Feeley I, Hughes AJ, Merghani K, Sheehan E, Kennedy M (2021). Injuries associated with arm wrestling: a narrative review. J Clin Orthop trauma.

[5] Rämö L, Taimela S, Lepola V, Malmivaara A, Lähdeoja T, Paavola M (2017). Open reduction and internal fixation of humeral shaft fractures versus conservative treatment with a functional brace: a study protocol of a randomised controlled trial embedded in a cohort. BMJ Open.

[6] Frankowska-Rutkowska M, Górska J, Jedwabiński M, Mątewski D, Maciejewski M (2013). Fracture of the humerus during arm wrestling: report on 9 cases and review of the literature. Med Biolog Sci.

[7] Ogawa K, Ui M (1997). Humeral shaft fracture sustained during arm wrestling: report on 30 cases and review of the literature. J Trauma.

[8] Whitaker JH (1977). Arm wrestling fractures-a humerus twist. Am J Sports Med.

[9] Kruczyński J, Jaszczur Nowicki J, Topoliński T (2012). Radiological and biomechanical analysis of humeral fractures occurring during arm wrestling. Med Sci Monit.

[10] Mayfield CK, Egol KA (2018). Humeral fractures sustained during arm wrestling: a retrospective cohort analysis and review of the literature. Orthopedics.

[11] Hong M-K, Lin C-Y, Liao Y-S, Hong C-K, Wang L-H (2022). Kinematic and electromyographic analysis of upper extremity in arm wrestling Accessed: July 19. https://ojs.ub.uni-konstanz.de/cpa/article/view/4824.

[12] Bumbaširević MŽ, Lešić AR, Andjelković SZ, Palibrk TD, Milutinović SM (2014). Fractures of the humerus during arm wrestling. Vojnosanit Pregl.

[13] Van Houwelingen A, McKee MD (2004). Management and complications of humeral shaft fractures. Univ Toronto Med J.

[14] An Z, Zeng B, He X, Chen Q, Hu S (2010). Plating osteosynthesis of mid-distal humeral shaft fractures: minimally invasive versus conventional open reduction technique. Int Orthop.

[15] Cole PA, Wijdicks CA (2007). The operative treatment of diaphyseal humeral shaft fractures. Hand Clin.

[16] Lin J, Hou SM (2003). Locked nailing of severely comminuted or segmental humeral fractures. Clin Orthop Relat Res.

